# Curcumin-Loaded Bamboo Shoot Cellulose Nanofibers: Characterization and In Vitro Studies

**DOI:** 10.3390/foods12183512

**Published:** 2023-09-21

**Authors:** Yu Chang, Qi Wang, Juqing Huang, Xianliang Luo, Yajuan Huang, Yirui Wu, Peng Chen, Yafeng Zheng

**Affiliations:** 1 College of Food Science, Fujian Agriculture and Forestry University, Fuzhou 350002, China; chang64131629@163.com (Y.C.); lxlsky008@163.com (X.L.); hyajuan2022@163.com (Y.H.); wyr516999@163.com (Y.W.); 18850609833@163.com (P.C.); 2 Institute of Agricultural Engineering, Fujian Academy of Agricultural Sciences, Fuzhou 350003, China; faaswq@163.com (Q.W.); jq_huang@zju.edu.cn (J.H.)

**Keywords:** cellulose nanofiber, bamboo shoot, curcumin, slow release

## Abstract

Given its high biological and pharmacological activities, curcumin (CUR) offers promising applications in functional foods. However, its low stability and bioavailability have greatly hindered its application in the food industry. The present study prepared cellulose nanofiber (CNF) from bamboo shoot processing byproducts and investigated its potential as a low-cost carrier. Our results showed that CUR was immobilized on CNF surfaces mainly through hydrogen bonding and eventually encapsulated in CNF matrices, forming a CNF–CUR complex with an encapsulation efficiency of 88.34% and a loading capacity of 67.95%. The CUR encapsulated in the complex showed improved stability after thermal and UV light treatments. Moreover, a slow and extended release pattern of CUR in a simulated gastrointestinal tract was observed, which could be appropriately described using the Korsmeyer–Peppas model. These results revealed that CNF is a promising protective carrier for the slow release of CUR, making it a better candidate for functional foods.

## 1. Introduction

Curcumin (CUR) is a biodegradable and biocompatible polyphenolic compound extracted from the root of the medicinal plant *Curcuma longa*. As one of the most intensively studied natural compounds, CUR has been shown to exhibit a wide range of biological and pharmacological activities, including anti-inflammatory, anticancer, antioxidant, and hypoglycemic effects, which are generated from the modulation of various biological targets, such as signaling pathways, transcription factors, inflammatory cytokines, enzymes, and apoptosis-related proteins [1,2,3,4]. After being approved by both the World Health Organization (WHO) and the Food and Drug Administration (FDA), CUR has been widely used as a food additive and natural pigment in the food industry to satisfy the rise in demand for healthy foods [5].

However, free CUR has a very low stability and is easily degraded under food processing and storage conditions. After oral administration, CUR exhibits poor bioavailability, meaning that only a small amount of CUR can survive, be absorbed in the gastrointestinal tract, and be released to the site of physiological activity. Recently, it has been assumed that CUR degradation products (e.g., vanillin, ferulic acid, and feruloylmethane) could also act as contributors, at least in part, to the various observed biological activities of CUR [6], while some studies have revealed contradictory observations, suggesting that the stable chemical degradation products of CUR are not likely to be responsible for the biological activities of CUR [7]. Although debatable, increasing clinical trial results have confirmed that employing various delivery carriers could improve the bioavailability of CUR, which corresponds to increased therapeutic efficacy [8]. Hence, developing suitable protective carriers for CUR is needed to overcome these drawbacks. With advancements in nanotechnology, various nanoformulations, like solid lipid nanoparticles [9,10], polymeric nanoparticles [11,12,13], nanoparticles [14,15,16], nanoemulsions [17,18], and nanocellulose [19], have been employed for the targeted and sustained release of therapeutics. However, complex preparation routines, high costs, and environmental risks may remain significant problems for researchers.

Cellulose nanofiber (CNF) is a nanometer-sized cellulosic product, which can be derived from abundant fiber materials, mainly agricultural product processing waste, through physical, chemical, or biological treatment [20]. CNF possesses the essential characteristics of cellulose as well as the properties of nanoparticles. Compared with ordinary dietary fibers, the unique nanostructures of CNF result in better rheology, water absorption, and mechanical properties. Previous studies have found that CNF exhibits good stability for use as a thickener in the food industry [21,22]. CNF also possesses high drug-encapsulating efficiency, a high surface-area-to-volume ratio, low cost, biocompatibility, biodegradability, environmental friendliness, and provides many binding sites for different compounds, including drugs [23], bioactive substances [24], and antibacterial agents [25], making it a promising candidate for constructing nanostructures and easing functionalization with respect to its application. Thus, CNFs have an undoubted advantage over other drug protective carriers due to their cost-effectiveness and ease of scalability, alongside all the mentioned advantages.

In our previous study, CNF was extracted from bamboo shoot processing byproducts, and it was found to exhibit improved properties compared to regular bamboo shoot dietary fiber [20]. Our results suggested that bamboo shoot CNF has great potential as a functional component or carrier in the food and pharmaceutical industries. Considering that both CUR and CNF are popular food ingredients in healthy food products, this study used bamboo shoot CNF as a novel and convenient protective carrier to encapsulate CUR to produce a new complex with improved stability and healthcare functions. Moreover, the prepared complex was characterized, and its stability and release profiles were also investigated.

## 2. Materials and Methods

### 2.1. Materials and Reagents

The CNF used in the present study was isolated from bamboo shoot (*Leleba oldhami* Nakal) processing byproducts (basal parts of the bamboo shoot) through low-concentration acid hydrolysis combined with ultrasonic treatment, which was reported in our previous study [20]. Briefly, the bleached bamboo shoot fiber was subjected to 5% (*v*/*v*) HCl solution at a ratio of 1:30 (*w*/*v*) and treated using an ultrasonic generator (KQ2200DE, Kunshan Ultrasonic Instrument, Shanghai, China) at an ultrasonic power of 170 W at 56 °C for 80 min. Then, the procedure was repeated under the same conditions, except for an increase in HCl concentration (10%, *v*/*v*). The resulting mixture was washed and freeze-dried to obtain a powder. The average length and diameter of the prepared nanofiber were 952.45 ± 38.53 nm and 69.97 ± 3.21 nm, respectively. The pure CUR was obtained from Shanghai yuan ye Bio-Technology Co., Ltd. Shanghai, China. All other reagents used were of analytical grade.

### 2.2. Preparation of the CNF–CUR Complex

**Figure 1:**
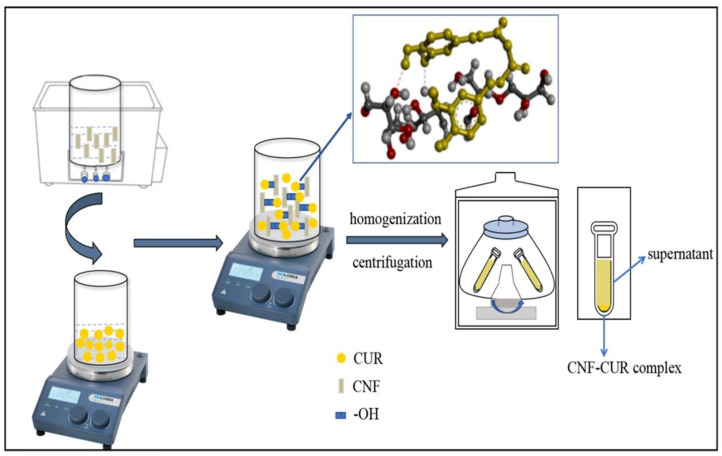
Schematic illustration of the formation of the CNF–CUR complex.

The preparation of the CNF–CUR complex was adapted from Li et al. [24] with modifications (Figure 1). Initially, 0.5 g of CUR was dissolved in 100 mL of 75% ethanol solution and stirred for 30 min at room temperature. Meanwhile, 30 mL of a CNF aqueous suspension (0.5%, *w*/*v*) was prepared, followed by 20 min of ultrasound treatment. The CNF aqueous suspension was added to the CUR ethanol solution, and the mixture was then stirred for 1 h to ensure adequate bonding between CUR and CNF. The mixture was then processed using a homogenizer (16,060× *g*) for 20 min and then centrifuged at 15,680× *g* for 20 min to obtain a pellet of the CNF–CUR complex. Finally, the samples were pre-frozen at −20 °C for 24 h and then placed in a freeze-dryer (Eyela 1200, Tokyo, Japan) and vacuum freeze-dried at −45 °C and 18 Pa to obtain freeze-dried powder samples, which were stored at 4 °C until further use.

### 2.3. Scanning Electron Microscopy (SEM)

The prepared sample was placed on a conductive adhesive, and the surfaces of the films were coated with a thin gold layer with a thickness of 0.01~0.1 μm using a vacuum coater. The cross-sectional morphology of each film sample was examined through observing the pretreated samples with an SEM (FEI QUANTATM 250, Thermo Fisher Scientific, Waltham, MA, USA) at 100,000× magnification with an acceleration voltage of 30 kV.

### 2.4. Encapsulation Efficiency and Loading Capacity

The determination of encapsulation efficiency (%) and loading capacity (%) of CUR was adapted from the reported method [23] with modifications. The freshly prepared CNF–CUR complex (0.1 g) was accurately weighed and dissolved in 50 mL of 95% (*v*/*v*) ethanol solution. The solutions were continuously stirred for 2 h and sonicated using the ultrasonic processor UP50H (Hielscher, Teltow, Germany, 50 W, 30 kHz) for complete dissolution.

The concentration of CUR in the solution was determined using a spectrophotometric method. The stock solution of CUR was serially diluted to obtain five different working concentrations, including 5, 10, 15, 20, 25, and 30 μg/mL of CUR, to establish a calibration curve. The absorbance was measured at 425 nm using an ultraviolet (UV)–visible spectrophotometer (UV-2000, UNICO, Shanghai, China). The regression equation was expressed as y = 0.008458x − 0.08556, R^2^ = 0.998.

The samples were centrifuged (12,040× *g*, 10 min), and the free CUR content in the supernatant was determined spectrophotometrically at 425 nm. The encapsulation efficiency and loading capacity of CUR can be calculated using the following equations:

$$Encapsulation\;efficiency\;(\%)=\frac{Initial\;CUR-Free\;CUR}{Initial\;CUR}\times 100$$

$$Loading\;capacity\;(\%)=\frac{Initial\;CUR-Free\;CUR}{Weight\;of\;the\;complex}\times 100$$

### 2.5. Fourier Transform Infrared (FT-IR) Spectroscopy

The comparative FT-IR spectra of CNF, CUR, and CNF–CUR were measured using FT-IR spectroscopy (VERTEX 70/70V, Bruker, Mannheim, Germany). Each sample (2%, *w*/*w*) was mixed with dry KBr, ground, and compressed into a KBr disk, which was subsequently scanned over a wavenumber range of 500–4000 cm^−1^.

### 2.6. Stability Measurements

The protective effect of CNF on CUR when exposed to UV light was evaluated using the method reported by Sun et al. [26] with minor modifications. In brief, freeze-dried powder of the CNF–CUR complex was dissolved in 10 mL of 50% (*v*/*v*) aqueous ethanol solution after mechanical stirring in order to prepare CNF–CUR complex sample solutions (1%, *w*/*v*). In accordance with the loading capacity of the CNF–CUR complex, free CUR solutions with equal concentrations to the encapsulated CUR in the complex were prepared and used as control. Freshly prepared sample solutions were placed in clear glass bottles and exposed to an ultraviolet light lamp (intensity of 0.35 W/m^2^) for 2, 4, 6, 24, and 48 h in a dark box at room temperature. The residual CUR content at different time points was determined spectrophotometrically. The concentration of CUR at 0 min was considered the initial concentration.

In addition, the heat stability of CUR loaded in CNF was evaluated according to a recently reported method [27]. Freshly prepared samples were placed in glass bottles and heated at 60 °C and 80 °C in a water bath for 30, 60, 90, 120, and 180 min, respectively. After the heat treatment, the samples were cooled to room temperature in an ice bath. Subsequently, the residual amount of CUR was determined. The retention rates of CUR after light or heat treatment were calculated using the following equation:

$$CUR\;retention\;(\%)=\frac{Residual\;CUR}{Initial\;CUR}\times 100$$

### 2.7. In Vitro Release Profile of CUR from CNF–CUR Complex

The in vitro release profile of CUR from the CNF–CUR complex under simulated gastrointestinal tract conditions was investigated using the dialysis bag method as reported by Fathi et al. [28] with adjustments. Briefly, 5 mL of CNF–CUR solution was mixed with 5 mL simulated gastric fluid (SGF) (2 g/L sodium chloride, 3.2 g/L pepsin, pH = 1.2) and placed in a dialysis bag in a beaker filled with 120 mL of the release medium (25% ethanol and 75% SGF without enzyme). The mixture was continuously shaken at 37 °C and 200 rpm. After 2 h, the pH of the solution was adjusted to 7 and then mixed with 5 mL of simulated intestinal fluid (SIF) (6 g/L potassium dihydrogen phosphate, 10 g/L pancreatin, 5 g/L bile salts, pH = 7.0). The dialysis bag was then placed in a beaker containing 120 mL of the release medium (25% ethanol and 75% SIF without enzyme) and shaken at 37 °C and 200 rpm for 4 h. At predetermined time intervals (0.5 h and 1 h), 1 mL sample solutions were removed from the release medium, and the same amount of fresh medium was added to the release medium. The concentration of released curcumin was determined spectrophotometrically at 425 nm.

### 2.8. Kinetic Study of In Vitro CUR Release

To gain insight into the release mechanism, three mathematical models, including the zero-order diffusion, Higuchi’s diffusion, and Korsmeyer–Peppas models, were utilized to predict the release behavior of CUR [29]. These models were useful for predicting the in vivo bioperformance of the prepared complex, and the best-fitted model was selected based on the highest correlation coefficient (R^2^) value.

### 2.9. Statistical Analysis

Statistical results were expressed as the mean ± standard deviation (S.D.) and performed in triplicate. DPS software (version 9.01) was used for statistical analyses. Analysis of variance (ANOVA) with Duncan’s range was used to compare the difference between groups, and *p* < 0.05 was considered significant.

## 3. Results

### 3.1. Characterization of CNF–CUR Complex

**Figure 2:**
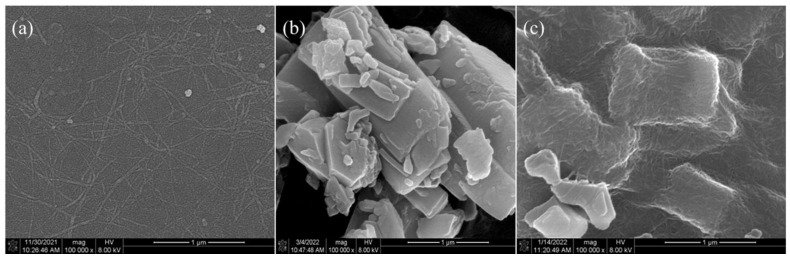
SEM images of the (**a**) CNF, (**b**) CUR, and (**c**) CNF–CUR complex.

SEM images of the CNF, CUR, and CNF–CUR complex are shown in Figure 2. CNFs with soft and long chain structures were haphazardly interconnected (Figure 2a), which may be due to the formation of H-bonds between the fibers [30]. The interconnected porous structure of CNF suggests that CNF could be a suitable candidate for loading and protecting bioactive substances [31]. The CUR showed irregular, lumpy structures of different sizes, and some lumps clustered together (Figure 2b). As observed in Figure 2c, after the encapsulation of CUR with CNF, most of the CUR was encapsulated with CNF. Meanwhile, a small part of curcumin was half-wrapped with CNF or distributed on the surface of the CNF, which might be due to a higher quantity of CUR, and a part of curcumin crystals were only adsorbed on the surface of CNF through H-bond interactions and Van der Waals forces.

Providing high entrapment efficiency and drug loading capacity is considered one of the most important advantages of a novel carrier. To check the suitability of the CNF–CUR complex as a carrier for CUR, the encapsulation efficiency and loading capacity of CUR were analyzed directly through measuring the amount of CUR in the suspension after the ethanolic extraction of CUR, which is a non-water-soluble polyphenol. According to the results, the CNF–CUR complex exhibited a promising encapsulation efficiency of 88.34% and a loading capacity of 67.95%.

### 3.2. FT-IR Analysis

**Figure 3:**
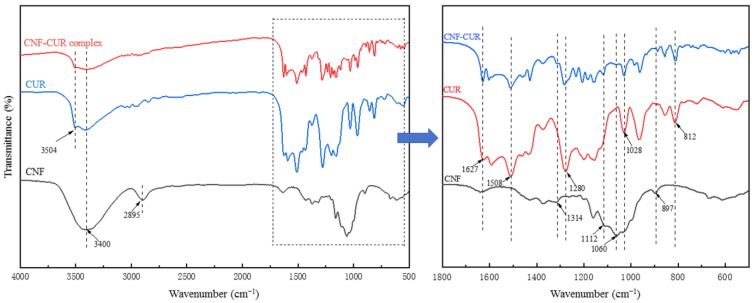
FT-IR spectra of the CNF, CUR, and CNF–CUR complex.

FT-IR spectra were analyzed in order to elucidate the chemical structure of the samples and the interactions between the CNF and CUR (Figure 3). The FT-IR spectra of the CNF presented a strong band around 3400 cm^−1^, assigned to the stretching vibration of the OH group associated with intermolecular hydrogen bonding. Meanwhile, the absorption peaks at 2895, 1430, and 1314 cm^−1^ were relative to CH vibration. The absorption peak at 1060 cm^−1^ was associated with the stretching vibration of the C-O-C bond on the pyran ring. The absorption peak at 897 cm^−1^ was attributed to the stretching vibration of the β-1, 4-glycosidic bond between cellulose molecules and the deformation vibration of O-H in the hydroxyl group of the sample that absorbed water molecules [32,33]. As for CUR, the broad absorption band at 3504 cm^−1^ represented a representative peak of the phenolic OH group’s stretching vibration. The peaks at 1627 and 1508 cm^−1^ were associated with the benzene ring stretching of C-H and C=C [30], and the absorption band at around 1028 cm^−1^ demonstrated the stretching vibration of C-O-C [34]. The characteristic peak at 812 cm^−1^ was associated with benzene ring vibration [35]. All characteristic peaks presented in the CNF and CUR spectra were also found in the FT-IR spectra of the CNF–CUR complex with slight shifts, suggesting the successful preparation of the complex. In comparison to the CUR spectrum, the intensity of the peaks at around 1627, 1508, 1280, 1028, and 812 cm^−1^ in the CNF–CUR complex spectrum, depicting C=O, C=C, and C-O-C stretching and benzene ring vibrations, decreased significantly, which could be due to the encapsulation of CUR onto the CNF through hydrogen bonding [24]. Another study has also reported that CUR can bind to cellulose through hydrogen bonding [30].

### 3.3. Improved Stability against UV and Heating

**Figure 4:**
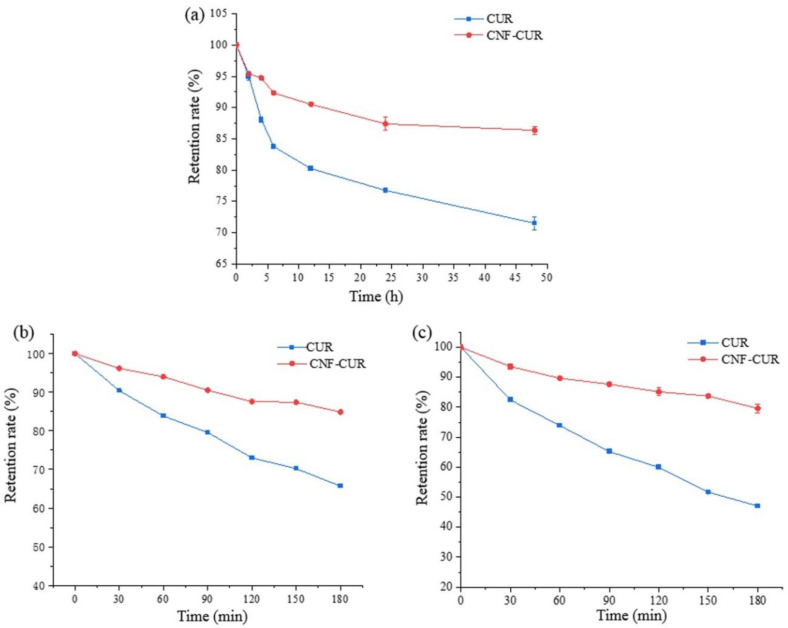
The retention rate (%) of CUR and CNF–CUR after UV irradiation (**a**) and heat treatment at 60 °C (**b**) and 80 °C (**c**).

CUR, both in liquid and solid states, is easily degraded under various environmental stresses, including light and heat. In the present study, the stability of CUR encapsulated in the complex under UV light and thermal treatments was determined and compared to that of free CUR. As shown in Figure 4a, after 48 h of UV light treatment, the retention rate of free CUR was quickly decreased to 71.04%. However, the encapsulated CUR in the complex was found to be less susceptible to UV light treatment, with a significantly higher retention rate of 86.21%, indicating that the encapsulation of CUR in carbohydrate polymers is an effective and feasible approach to strengthen its photochemical stability. This improvement in photochemical stability is likely due to intermolecular hydrogen bond formation between CUR and CNF molecules.

The CNF–CUR complex and free CUR were heated at 60 °C and 80 °C for 180 min in a water bath to evaluate the effect of complexation with CNF on the thermal stability of CUR (Figure 4b,c). It was observed that the degradation rate of CUR was faster than that of the CNF–CUR complex, whether heated at 60 °C or 80 °C. After 180 min of heat treatment at 80 °C, the content of free CUR decreased to 47.02%, while the retention rate of CUR in the CNF–CUR complex was as high as 79.58%. In the complex, CUR distributed on the surface could still be degraded through thermal treatment. This significantly improved thermal stability of CUR is mainly due to the encapsulated CUR inside the CNF matrix, which provides sufficient protection for the CUR under thermal stress [36].

### 3.4. In Vitro Release Profile and Release Kinetics of CUR from the Complex

**Figure 5:**
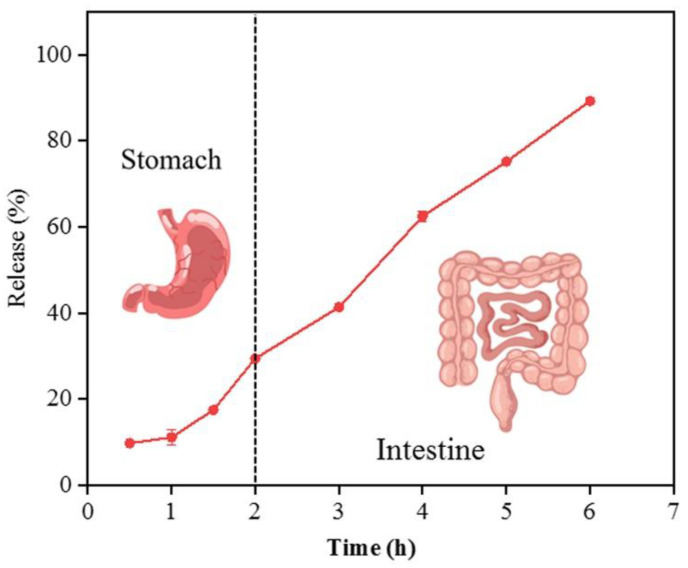
Release of CUR from the complex in the simulated gastrointestinal environment.

Understanding the release profile of encapsulated CUR plays a crucial role in determining the efficacy of the CNF–CUR complex and its potential application in functional foods. The release profile of CUR from the complex was investigated in a simulated gastrointestinal environment. As depicted in Figure 5, during incubation in the simulated gastric digestion conditions, only 10.12% and 29.39% of CUR was slowly released from the CNF–CUR complex at 1 h and 2 h, respectively. This sustained slow release of CUR could be related to the matrix network of CNF and strong hydrogen bonding that suppressed the release of CUR. It has been reported that nanoparticles can be used as carriers to protect CUR against acid degradation in the simulated stomach environment and deliver most CUR to the intestine, contributing to improved bioavailability [31]. Further, during incubation in simulated intestinal digestion conditions for 4 h, a similar release rate of CUR from the complex was observed, and 82.83% of CUR was released after 6 h of simulated gastrointestinal digestion. Hence, these results suggest that being encapsulated into CNF could inhibit the burst release of CUR in the stomach environment and enable the sustained release of most CUR into the intestine. For oral delivery purposes, this slow release of CUR in gastrointestinal conditions is required because a higher amount of bioactive compounds is available for intestinal adsorption [37].

**Figure 6:**
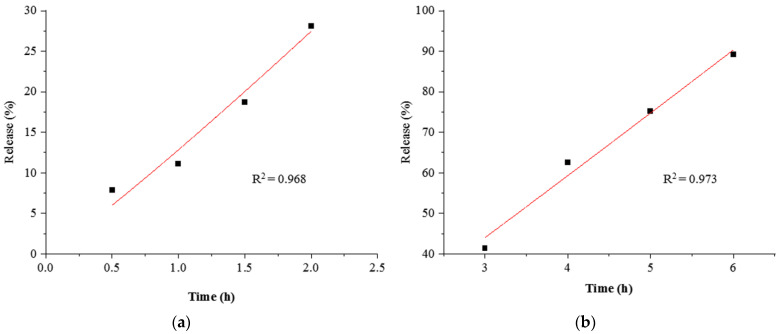
Release kinetics evaluation of CUR from the CNF–CUR complex in the simulated stomach (**a**) and intestine (**b**) using the Korsmeyer–Peppas model.

The mechanism behind CUR release from the complex in gastrointestinal conditions was analyzed using various kinetic equations, including the Korsmeyer–Peppas, zero-order, and Higuchi models. As shown in Table 1, the Korsmeyer–Peppas model could adequately describe the release of encapsulated CUR from the complex in gastrointestinal conditions, with the highest R^2^ values of 0.968 (stomach) and 0.973 (intestine). The fitted equation graphs of CUR release are represented in Figure 6. In the Korsmeyer–Peppas model, the n parameter is used as the diffusional exponent to elucidate the ingredient transport mechanism. The n < 0.45 represents Fick’s diffusion mechanism, and 0.45 < n < 0.89 indicates the non-Fickian diffusion mechanism [38]. When the value of n is larger than 1, the release mechanism is regarded as Super Case-II transport or zero-order release kinetics [23,39].The n values for CUR release were found to be 1.09 and 1.03, respectively, suggesting that CUR release from the complex in gastrointestinal conditions occurred through diffusion, swelling, and CNF relaxation due to erosion of the polymer [23].

## 4. Discussion

CUR is widely applied in various healthy food products. However, its biological function is significantly restricted by its high sensitivity to light, pH, and heat during food processing and storage. Moreover, as most CUR products are administered orally, CUR is easily destroyed during the gastrointestinal digestive process, further limiting its bioavailability. Therefore, a suitable protective carrier should be used for the encapsulation of CUR to increase its stability and preserve its health-promoting properties. Our previous study showed that CNF prepared from bamboo shoot exhibits promising physicochemical properties, indicating that it is a suitable functional component or carrier in food and pharmaceutical industries. Taking advantage of the high adsorption efficiency and high surface-area-to-volume ratio of CNF, this study used CNF to encapsulate CUR into its assembly matrices. The binding of CUR onto the surface of the CNF was confirmed through morphological analysis and the FT-IR technique. It was found that most CUR may be enclosed or half-enclosed in the CNF matrix through hydrogen bonding, and only a small part of CUR was adsorbed on the surface of the CNF. Encapsulation efficiency is one of the most important factors for evaluating new carriers. The CNF–CUR complex prepared in this study had an encapsulation rate of 88.34%, indicating that CUR was efficiently protected in the developed complex. The encapsulation rate of CUR (88.34%) in this study is comparable to the values of recently reported CUR-loaded carries, including chitosan/poly (ε-caprolactone) nanoparticles (70.9%) [40], whey protein-based nanoemulsions (90.56%) [41], *Lallemantia iberica* seed gum (LISG)/Ca^2+^ nanoparticles (NPs, 88.23%), and LISG/chitosan NPs (97.3%) [28]. More importantly, among these approaches, the creation of the CNF–CUR complex is promising due to its simple preparation, low cost, nontoxicity, and biodegradability, especially for industrial applications.

The stability of CUR can deteriorate during food processing and storage because of its sensitivity to light and heat. The results of the stability test showed that CNF could significantly improve the photochemical and thermal stability of CUR under environmental conditions. After UV light irradiation, compared to the retention rate of free CUR (71.04%), the encapsulated CUR in the complex was found to have a significantly higher retention rate of 86.21%, indicating that only 13.79% of CUR was degraded. In another similar study, Sun et al. [26] evaluated the effect of complexation with linear dextrin on curcumin photochemical stability and found that only 11~18% of curcumin complexed with linear dextrin degraded after UV irradiation, while 32% of free curcumin degraded under the same conditions. These results indicate that the encapsulation of CUR in carbohydrate polymers is an effective and feasible approach to strengthen its photochemical stability. As heat treatment is one of the most important processes in food preparation, it is of great significance to evaluate the thermal stability of CUR. Our results indicate that the encapsulation of CUR in the complex is an effective and feasible approach to strengthen its thermal stability. Due to the high thermal stability and strong binding capacity of CNF, the encapsulation of CUR within the CNF matrix could protect the chemically active groups of CUR from thermal degradation. Meanwhile, the formation of hydrogen bonds between CUR and CNF is conducive to changing the microstructure and improving thermal and UV light stability [27]. Similar results have also been reported when a glycated soy β-conglycinin nanoparticle was developed as a CUR-loaded carrier to improve its thermal stability [42].

In the simulated gastrointestinal environment, our results demonstrated a slow and extended release pattern of CUR from the complex, thereby reducing the decomposition of CUR in the acidic environment of the stomach and promoting the slow release and absorption of CUR in the small intestine. At the same time, part of the CUR and CNF might enter the large intestine, exerting synergistic health effects on the microbiota. In addition, the release kinetics of the CNF–CUR complex was evaluated using different release kinetics equations, and it was found that the Korsmeyer–Peppas model could appropriately describe the release of CUR. Overall, our results suggest that CNF prepared from bamboo shoot processing byproducts could be used as not only a novel dietary fiber, but also a promising carrier system to improve the stability and bioavailability of CUR.

## 5. Conclusions

The present study applied a simple and low-cost method to prepare a CUR–CNF complex with a CUR encapsulation efficiency of 88.34%. CUR encapsulated in the complex showed improved stability under heat and light treatments and exhibited a slow and extended release pattern in a simulated gastrointestinal environment. Based on these promising in vitro results from the present work, the health effects of the CUR–CNF complex need to be further investigated using an animal model, and the underlying mechanism should also be clarified.

## Figures and Tables

**Table 1:** Modeling of CUR release kinetics from CNF in the stomach and intestine.

Medium	Model	R^2^	Adj-R^2^	RMSE	k	n
Stomach	Zero-order	0.805	0.787	1.636	0.417	-
Intestine	Zero-order	0.870	0.858	0.560	0.311	-
Stomach	Higuchi	0.941	0.935	0.498	2.654	-
Intestine	Higuchi	0.970	0.968	0.128	1.932	-
Stomach	Korsmeyer–Peppas	0.968	0.965	0.266	9.378	1.09
Intestine	Korsmeyer–Peppas	0.973	0.971	0.115	8.968	1.03

## Data Availability

Not applicable.
